# Comparative evaluation of an improved test method for bioefficacy of insecticidal fabrics against dengue and malaria vectors

**DOI:** 10.1186/s13071-019-3637-y

**Published:** 2019-07-29

**Authors:** Reji Gopalakrishnan, Avik Mazumder, Ruchi Yadav, Damayanti Meher, Ram Singh, Devanathan Sukumaran, Vikas B. Thakare, Meehir Palit

**Affiliations:** Defence Research & Development Establishment, Gwalior, 474002 India

**Keywords:** Insecticidal fabrics, Permethrin, Disease vector, Bioefficacy test

## Abstract

**Background:**

Insecticidal fabrics are important personal protective measures against mosquitoes, ticks and other disease vectors. In the absence of internationally accepted guidelines, bioefficacy tests have been carried out using continuous exposure and three minutes exposure bioassay methods. Recently, we have reported an improved method for bioefficacy testing of insecticidal fabrics, which involves continuous exposure of mosquitoes to the test fabrics. The present paper reports the comparative evaluation of the outcomes of the continuous exposure bioassay and the three minutes bioassay on the same fabric samples.

**Methods:**

Permethrin content in the treated fabric samples was determined through HPLC analysis and NMR studies were performed to establish the stability of the analyte. Bioefficacy tests were carried out against dengue vector *Aedes aegypti* and malaria vector *Anopheles stephensi* as per the improved test method and the three minutes bioassay method.

**Results:**

The permethrin doses in the fabric samples ranged from 60 to 3000 mg/m^2^ and 36.2% of permethrin was retained after 10 washings. The extraction and chromatographic analysis were not found to affect the stability of permethrin. In continuous exposure, all fabric samples showed bioefficacy, as the mean complete knockdown time for both *Ae. aegypti* (10.5–34.5 min) and *An. stephensi* (14.5–36.8 min) was ≤ 71.5 min. The same samples were found to be not effective when tested using the three minutes bioassay method, since the knockdown and mortality percentages were well below the required bioefficacy values. The bioefficacy of the fabric samples in terms of complete knockdown time was significantly higher against *Ae. aegypti* in comparison to *An. stephensi*. The mean complete knockdown time of *Ae. aegypti* increased to 48.3 min after 10 washings indicating a significant reduction in bioefficacy.

**Conclusions:**

Bioefficacy testing of the insecticidal fabrics using the improved method resulted in outcomes, which could be correlated better with the permethrin content in the fabric samples. The improved method is more appropriate for the testing of insecticidal fabrics than the three minutes bioassay method. Further evaluation of the improved method using different test arthropods could help in the formulation of specific guidelines for the bioefficacy testing of insecticidal fabrics.

## Background

The geographical range of *Aedes* mosquitoes, such as *Ae. aegypti* and *Ae. albopictus*, and the pathogens transmitted by them are expanding at a fast pace [1]. Originally restricted to tropical and subtropical regions, these mosquitoes now have a worldwide distribution [2] and spread arboviral diseases including dengue, Zika, chikungunya and yellow fever [3]. Currently, dengue has emerged as a major global public health problem with millions of cases reported every year [4–6]. Malaria is another major vector-borne disease with a global distribution and is transmitted by *Anopheles* mosquitoes. *Anopheles stephensi* is an important vector of malaria in India, and is prevalent in many parts of Asia and the Middle East [7]. Dengue, malaria and other mosquito-borne diseases cause substantial loss of workdays and financial loss across the world [1]. Novel vector control measures such as the use of *Wolbachia* are under development but not yet available for routine use [8, 9]. Prevention of arthropod bites by the use of insecticide-treated clothes and bednets is one of the most effective methods for prevention of vector-borne infections. At present, long-lasting insecticidal nets (LLIN) containing synthetic pyrethroid insecticides are widely used for malaria control across the world. However, these nets cannot provide protection from *Aedes* mosquitoes, which are active during the daytime. This necessitates the use of products suitable for daytime use such as insecticidal fabrics [3]. Such treated clothing can protect the user from the bites of day and night biting mosquitoes in both indoor and outdoor environments. Moreover, the use of LLIN may become impractical during natural disasters, political conflicts or in refugee camps [10]. In some malaria endemic regions of the world, socioeconomic factors such as lack of proper housing, lack of sleeping facilities, non-affordability of bednets and outdoor sleeping habits lead to non-ownership or non-usage of bed nets [11]. In such scenarios, treated clothing or blankets could reduce infectious disease burden on displaced and vulnerable populations [11, 12]. Fabric treatment with the insecticide permethrin has a long history and has been practised by the armed forces of many countries including the USA, Australia, Germany and France [13–16]. Insecticidal fabrics are now commercially available for civilian use as well [17] and various studies over the years have shown the bioefficacy of these fabrics against mosquitoes and other biting arthropods [18, 19].

Insecticidal fabrics factory treated with permethrin reportedly retain bioefficacy even after repeated wash-dry cycles [16, 17, 20]. However, such claims on the long-lasting bioefficacy of insecticidal fabrics need to be verified using an internationally accepted test method. At present, there are no international guidelines for the testing of insecticidal fabrics [16, 18, 19], which makes the comparison of the performance of different products in laboratory trials a daunting task. WHO guidelines are available for bioefficacy testing of LLIN [21] but the suitability of these guidelines for the testing of insecticidal fabrics is yet to be ascertained. Guidelines specific to insecticidal fabrics are needed since the insecticidal fabrics and LLIN differ considerably in their method of use, method of treatment, type of fibres and the frequency of washing [19]. In this context, we have formulated an improved test method for the bioefficacy of insecticidal fabrics [19], which could be used until internationally accepted guidelines are available. This method was formulated based on the TL 8305-0331 test protocol of the German Armed Forces [16, 17] and the WHO guidelines on bioefficacy testing of LLIN [21] with suitable modifications. The improved test method involves continuous exposure of mosquitoes to insecticidal fabric samples. Five mosquitoes are introduced into a standard WHO cone fixed over the test sample. The mosquitoes are held in the cone until all five test mosquitoes are knocked down. The time required to achieve complete (100%) knockdown is recorded as the complete knockdown time (CKDT). The tests are replicated ten times. The test fabrics are effective if the mean CKDT is  ≤ 71.5 minutes [19].

Further assessment of the validity of the improved method through testing against major disease vectors and the correlation of the bioefficacy results with the permethrin content would be helpful to ensure the suitability and robustness of the method. As per the improved method and the TL 8305-0331 protocol, the mosquitoes are continuously exposed to insecticidal fabrics and the time required for knockdown is measured. However, the WHO method for LLIN relies on the mosquitoes being exposed to the test samples for three minutes and the establishment of percent knockdown and mortality. A comparative evaluation of the outcomes of the continuous and the three minutes exposure methods on the same fabric samples is required. Such a study would provide valuable insights on the relative performance of these two test methods and strengthen our efforts towards the standardisation of test methods for insecticidal fabrics.

The objective of the present study was to compare the outcomes of the improved test method and the three minutes bioassay method on the same fabric samples. This would help us to understand whether the insecticidal fabric samples found effective as per one test method would be effective as per the other test method or not. Additionally, the study aimed to compare the susceptibility of dengue vector *Ae. aegypti* with *An. stephensi*, which is an important vector of malaria in India. The bioefficacy results were correlated with the permethrin residues quantified through high performance liquid chromatography (HPLC). The stability of permethrin during the extraction and chromatographic procedures was studied through nuclear magnetic resonance (NMR) analysis.

## Methods

### Test fabric

Polyester-cotton blend (20:80) military uniform fabrics were used in the study. A series of eight concentrations of technical grade permethrin (cis:trans 25:75; Tagros Chemicals India Ltd., Chennai, India) were prepared in isopropanol and the fabrics were dipped in these solutions for 3 h. The treated fabric samples were coded A to H, A having the highest dose and H having the lowest dose. Samples (15 × 15 cm) of the treated fabrics were used for bioefficacy testing whereas untreated fabrics were used as control.

### Sample preparation and HPLC analysis

The fabric samples (25 mg) were subjected to solvent extraction using 1 ml of acetonitrile for 45 min at 30 °C in a Branson 2510 ultrasonic bath (Branson Ultrasonics, North Billerica, MA, USA). The extract was injected into an Agilent 1100 high performance liquid chromatography (HPLC) system (Agilent Technologies, Waldbronn, Germany) equipped with a Rheodyne injector (loop volume 20 µl) and a variable wavelength UV detector (UV-VWD at 225 nm). Chromatographic separation was achieved at 40 °C using Zorbax Extend-C_18_ HPLC column (4.6 × 150 mm, 5 µm; Agilent Technologies, Santa Clara, CA, USA) at a flow rate of 1.2 ml/min using water and acetonitrile gradient elution.

### NMR analysis

In order to evaluate the stability of permethrin, nuclear magnetic resonance (NMR) experiments were performed using technical grade permethrin before and after ultrasonication at 30 °C for 45 min. All experiments were performed under non-spinning mode on a Bruker AVIII 600 NMR spectrometer (Bruker BioSpin, Fällanden, Switzerland) equipped with a BBFO 5 mm NMR probe (temperature: 25 ± 1 °C; air flow rate: 400 l/min). The data acquisition and processing were performed using Topspin v.3.5pl7 software. The standard pulse sequence *zg* found in the pulse program library was used for this purpose.

### Washing

The treated fabrics were washed in the laboratory as per the WHO standard washing procedure for LLIN [21]. The fabric samples (25 × 25 cm) were introduced into a beaker containing 2 g/l soap solution in deionised water. The beaker was then kept for 10 min in a water bath shaker a 155× *rpm* at 30 °C. This was followed by rinsing in deionised water twice for 10 min each. The washed samples were dried under shade at room temperature. The fabric samples were washed 10 times with an interval of 24 h between successive washings.

### Test insects

*Aedes aegypti*, a major vector of dengue and *Anopheles stephensi*, an important vector of malaria in India, were used as the test insects. The mosquitoes were obtained from the insect rearing facility at Defence Research and Development Establishment (DRDE), Gwalior, India. The mosquitoes were maintained at a temperature of 27 ± 2 °C and a relative humidity of 70 ± 10%. Two- to five-days-old, non-blood-fed adult female mosquitoes were used for the tests.

### Continuous exposure bioassay

The test fabrics were subjected to bioefficacy testing as per the improved test method [19]. The method involved continuous exposure of mosquitoes to the insecticidal fabric samples. Five mosquitoes were introduced into a standard WHO cone fixed over the test sample. The test mosquitoes were held in the cone until all the five mosquitoes were knocked down. The time required to achieve complete (100%) knockdown of the test mosquitoes was recorded as the complete knockdown time (CKDT). The tests were replicated ten times. The test fabrics were effective if the mean CKDT was ≤ 71.5 min.

### Three minutes bioassay

Three minutes exposure bioassays were conducted as per the WHO guidelines for testing of LLIN [21]. Five mosquitoes were introduced into a standard WHO cone fixed over the test sample for 3 min. Thereafter, the mosquitoes were removed from the cone and released into a plastic bowl covered with gauze. The percent knockdown 1 h post-exposure (KD) and the percent mortality 24 h post-exposure (MR) of the test mosquitoes were recorded. The mosquitoes were given access to 10% sucrose solution during the 24 h observation period. The tests were replicated 10 times. The mosquitoes exposed to untreated fabric samples served as the control. The test fabrics are effective if the mean percent knockdown was ≥ 95% and/or the mean percent mortality was ≥ 80%.

### Statistical analysis

One-way analysis of variance (ANOVA) followed by Tukey’s HSD was used to analyse the difference in the bioefficacy of the fabric samples containing different permethrin doses and after repeated washings. The susceptibility of *Ae. aegypti* and *An. stephensi* mosquitoes to the fabric samples containing different doses of permethrin were compared using a t-test.

## Results

### HPLC and NMR analyses

**Fig. 1:**
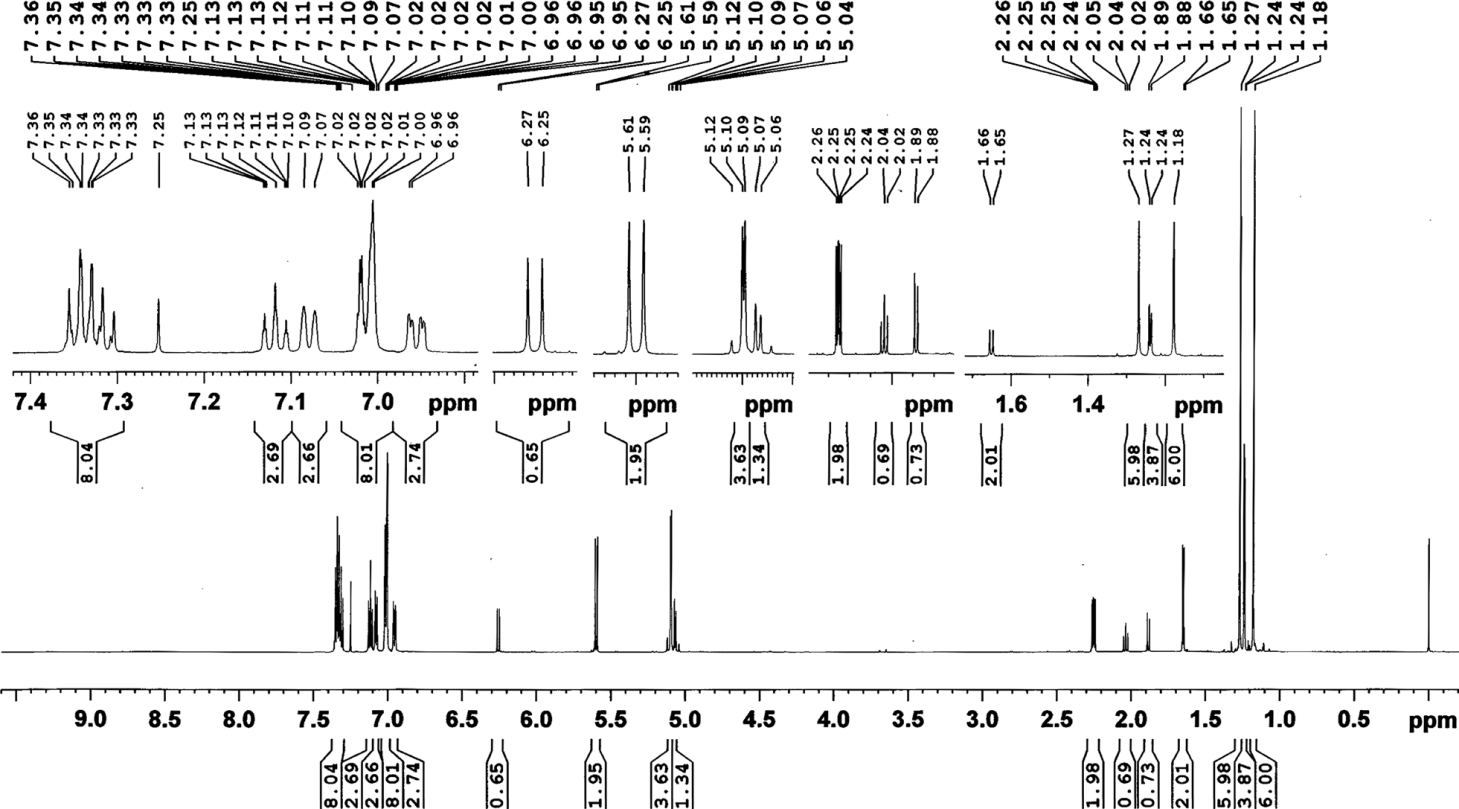
^1^H-NMR spectra (25 °C) of technical grade permethrin (cis:trans 25:75) in CDCl_3_

The permethrin content in the fabric samples A to H were 3000, 2280, 1590, 1040, 590, 380, 170 and 60 mg/m^2^, respectively (Table 1). Only sample C, with a permethrin dose (1590 mg/m^2^) closest to the recommended maximum dose of 1600 mg/m^2^, was subjected to repeated washings. The permethrin residues in sample C after 1 to 10 washings were 1353, 1051, 921, 911, 891, 770, 710, 689, 644 and 576, respectively. The percent retention of permethrin in sample C after the first wash was 85.1, which dropped to 56 after the fifth and to 36.2 after the tenth washing (Table 2). The NMR experiments clearly showed that new signals were not generated nor was there a significant shift or change in the shape of the peaks that were originally observed when the spectra were recorded in CDCl_3_ (non-reactive solvent) (Fig. 1). These experiments indicated that the analyte (permethrin) was not degraded during the extraction.

**Table 1:** Bioefficacy of insecticidal fabric samples containing different doses of permethrin against *Aedes aegypti* and *Anopheles stephensi* mosquitoes in continuous exposure bioassay (improved test method) and three minutes exposure bioassay (WHO test method for LLIN)

Sample	Permethrin dose (mg/m^2^)	*Aedes aegypti*			*Anopheles stephensi*		
		Continuous exposure mean CKDT in min (95% CI)	Three minutes exposure		Continuous exposure mean CKDT in min (95% CI)	Three minutes exposure	
			Mean KD% (95% CI)	Mean MR% (95% CI)		Mean KD% (95% CI)	Mean MR% (95% CI)
A	3000	10.5^a^ (10.0–11.0)	86^c^ (75.8–96.2)	72^c^ (53.3–90.7)	14.5^a^ (13.6–15.4)	76^d^ (64.6–87.4)	48^c^ (39.3–56.7)
B	2280	11.4^a^ (10.2–12.6)	74^bc^ (59.6–88.4)	64^bc^ (47.7–80.3)	14.8^a^ (14.3–15.3)	68^cd^ (54.7–81.3)	42^bc^ (29.7–54.3)
C	1590	11.8^a^ (10.5–13.1)	70^bc^ (59.5–80.5)	52^abc^ (37.4–66.6)	15.3^a^ (13.9–16.7)	60^cd^ (49.9–70.1)	36^abc^ (24.6–47.4)
D	1040	12.5^ab^ (11.7–13.3)	62^bc^ (44.0–80.0)	46^abc^ (26.6–65.4)	17.7^ab^ (16.3–19.1)	44^bc^ (27.7–60.3)	30^abc^ (14.3–45.7)
E	590	14.8^bc^ (14.1–15.5)	58^bc^ (41.0–75.0)	40^abc^ (25.7–54.3)	22^bc^ (19.8–24.2)	26^ab^ (12.9–39.1)	26^abc^ (9.42–42.6)
F	380	15.8^c^ (15.3–16.3)	48^ab^ (33.4–62.6)	32^ab^ (20.0–44.0)	24.5^cd^ (22.2–26.8)	20^ab^ (8.3–31.7)	20^ab^ (9.88–30.1)
G	170	22.8^d^ (20.3–25.3)	44^ab^ (29.9–58.1)	22^a^ (5.0–39.0)	29.7^d^ (25.8–33.6)	12^a^ (0–24.0)	16^ab^ (6.22–25.8)
H	60	34.5^e^ (33.6–35.4)	26^a^ (15.8–36.2)	20^a^ (4.5–35.5)	36.8^e^ (31.9–41.7)	10^a^ (1.2–18.8)	12^a^ (1.55–22.5)
ANOVA		*F*_(7, 72)_ = 168.8, *P* < 0.001	*F*_(7, 72)_ = 7.15, *P* < 0.001	*F*_(7, 72)_= 5.21, *P *< 0.001	*F*_(7, 72)_ = 36.7, *P* < 0.001	*F*_(7, 72)_ = 17.4, *P* < 0.001	*F*_(7, 72)_ = 4.17, *P* = 0.001

*Note*: Means followed by the same letters in a column are not significantly different (*P*>0.05) in ANOVA followed by Tukey’s HSD

*Abbreviations*: CKDT, complete knockdown time; CI, confidence interval; KD, knockdown; MR, mortality

**Table 2:** Permethrin residues on insecticidal fabric after repeated washings and bioefficacy against *Aedes aegypti* mosquitoes in continuous exposure bioassay (improved test method)

Wash no. (Sample C)	Permethrin residue (mg/m^2^)	Retention of permethrin (%)	Continuous exposure mean CKDT in min (95% CI)
0	1590	100	10.5^a^ (10.0–11.0)
1	1353	85.1	16^ab^ (14.4–17.6)
2	1051	66.1	17.3^abc^ (14.9–19.7)
3	921	57.9	18.8^abc^ (17.2–20.4)
4	911	57.3	22.3^bc^ (20.7–23.9)
5	891	56.0	25.3^c^ (23.8–26.8)
6	770	48.4	36^d^ (32.4–39.6)
7	710	44.7	39.5^de^ (35.6–43.4)
8	689	43.3	42.4^de^ (38.3–46.5)
9	644	40.5	44.8^de^ (37.5–52.1)
10	576	36.2	48.3^e^ (41.2–55.4)

*Note*: Means followed by the same letters in a column are not significantly different (*P* > 0.05) in ANOVA (*F*_(10, 99)_ = 45.5, *P *< 0.001) followed by Tukey’s HSD

*Abbreviations*: CKDT, complete knockdown time; CI, confidence interval

### Bioefficacy

In the continuous exposure bioassay as per the improved method, the CKDT of *Ae. aegypti* mosquitoes against the sample A (highest dose) was 10.5 min. The CKDT increased to 11.4, 11.8, 12.5, 14.8, 15.8, 22.8 and 34.5 min for the samples B to H, respectively. There was a significant increase in CKDT due to the decrease in permethrin dosage (*F*_(7, 72)_ = 168.8, *P *< 0.001).In the three minutes exposure bioassay, the mean percent knockdown (KD) decreased significantly from 86 for sample A to 26 for sample H (*F*_(7, 72)_ = 7.15, *P *< 0.001) whereas the mean percent mortality (MR) decreased significantly from 72 for sample A to 20 for sample H (*F*_(7, 72)_ = 5.21, *P *< 0.001) (Table 1).

The CKDT of *An. stephensi* mosquitoes against sample A (highest dose) was 14.5 min in the continuous exposure bioassay. CKDT increased to 14.8, 15.3, 17.7, 22, 24.5, 29.7 and 36.8 min for the samples B to H, respectively. There was significant increase in CKDT due to the decrease in the permethrin dose (*F*_(7, 72)_ = 36.7, *P *< 0.001).The percent KD in the three minutes exposure bioassay decreased significantly from 76 for sample A to 10 for sample H (*F*_(7, 72)_ = 17.4, *P *< 0.001) whereas the percent MR decreased significantly from 48 for sample A to 12 for sample H (*F*_(7, 72)_ = 4.17, *P* = 0.001).

### Differential susceptibility

**Fig. 2:**
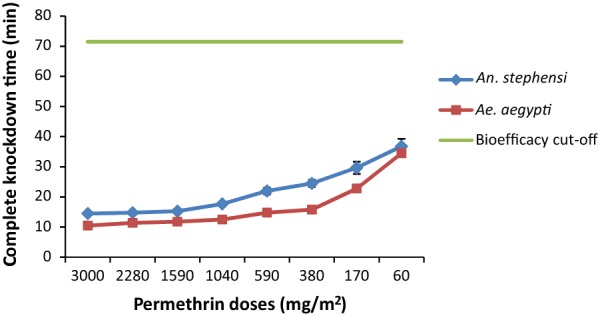
Differential susceptibility of *Aedes aegypti* and *Anopheles stephensi* mosquitoes on continuous exposure to insecticidal fabrics (error bars represent the standard error of the mean)

**Fig. 3:**
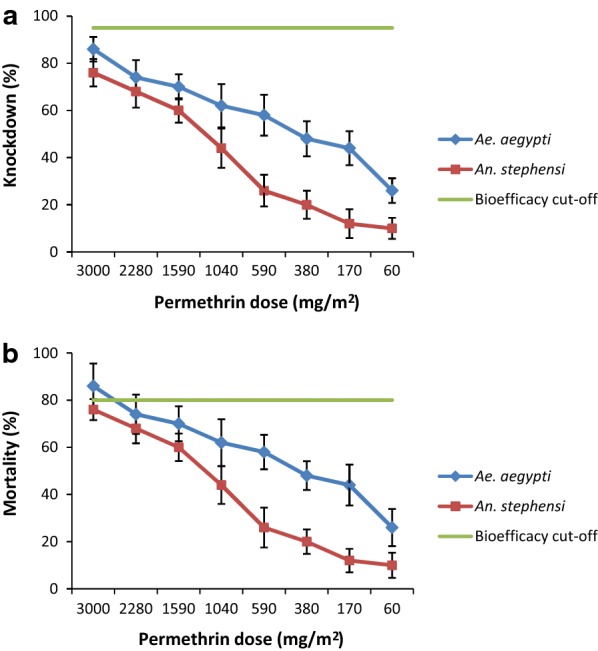
Differential susceptibility of *Aedes aegypti* and *Anopheles stephensi* mosquitoes to 3 min exposure to insecticidal fabrics. **a** Percent knockdown. **b** Percent mortality. Error bars represent the standard error of the mean

The differences in the susceptibility of *Ae. aegypti* and *An. stephensi* mosquitoes against different permethrin doses were compared. The CKDT of *Ae. aegypti* was significantly lower than *An. stephensi* for all samples (A: *t*_(9)_ = 8.48, *P *< 0.001; B: *t*_(9)_ = 5.85, *P *< 0.001; C: *t*_(9)_ = 5.50, *P *< 0.001; D: *t*_(9)_ = 6.19, *P *< 0.001; E: *t*_(9)_ = 5.66, *P *< 0.001; F: *t*_(9)_ = 6.65, *P *< 0.001; G: *t*_(9)_ = 2.68, *P* = 0.025) except for sample H (*t*_(9)_ = 0.95, *P* = 0.369) (Fig. 2). In the three minutes exposure bioassay, the percent KD of *Ae. aegypti* was higher in comparison to *An. stephensi* for all samples tested but the difference was not significant except for samples E, F and G (Fig. 3a). Similarly, a higher MR of *Ae. aegypti* was recorded for all samples but the difference was significant only for sample B (Fig. 3b).

### Washing

**Fig. 4:**
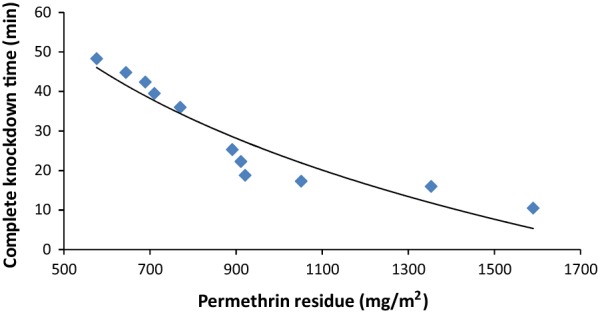
Permethrin residue in insecticidal fabric after repeated washings and the corresponding complete knockdown time of *Aedes aegypti* mosquitoes

The CKDT of *Ae. aegypti* against the sample C increased from 10.5 min before washing to 16, 17.3, 18.8, 22.3, 25.3, 36, 39.5, 42.4, 44.8 and 48.3 after 10 standard washings. The increase in CKDT due to washing was significant (*F*_(10, 99)_ = 45.5, *P *< 0.001) (Table 2). The change in the CKDT in relation to the decrease in the permethrin residues due to repeated washing is shown in Fig. 4.

## Discussion

Insecticidal fabrics can serve as effective barriers against disease vectors and their use has emerged as a potential public health intervention for protection from day biting mosquitoes transmitting dengue, chikungunya and Zika viruses [3]. Various studies across the world have demonstrated the ability of insecticidal fabrics to protect from arthropod bites [18, 22, 23]. Currently, there are no international guidelines on insecticidal fabrics [16, 18] which makes the comparison of laboratory and field performance of different products challenging. As per the WHO guidelines, cone bioassays with three minutes exposure are used for bioefficacy testing of LLIN wherein the nets providing at least 95% mosquito KD or 80% MR are deemed to be effective [21]. However, adequate information is not yet available on the suitability of this method for the testing insecticide treated military uniforms and other clothes.

The German Armed Forces (Bundeswehr) relies on the TL 8305-0331 testing protocol for the testing of battle dress uniforms (BDU) treated with permethrin [16, 17]. As per this method, the test mosquitoes are continuously exposed to the treated cloth samples and the time of exposure necessary to obtain 99% KD (“KD_99_”) is recorded. The cloth samples, which have a “KD_99_” of not more than 71.5 minutes are declared to be effective as per this test method [16, 17]. Whether the treated clothes found effective using this test method fulfil the bioefficacy criteria as per the three minutes exposure method or *vice versa* is yet to be confirmed. This could be ascertained only through the evaluation of the same treated cloth sample using the continuous exposure and the three minutes exposure methods. In this context, the present study is, to our knowledge, the first report on the comparative evaluation of cloth samples treated with different doses of permethrin using continuous and three minutes exposure methods. The results of the present study have shown that all the eight test samples with permethrin doses in the range of 60–3000 mg/m^2^ passed the bioefficacy criteria of the improved test method. However, all these samples failed to meet the bioefficacy criteria of the three minutes bioassay method. Thus, it is clear that much higher doses of permethrin would be required for the fabrics to qualify the efficacy testing as per the three minutes bioassay method. Such samples with high doses of permethrin would obviously meet the bioefficacy criteria of the improved test method as well. However, permethrin doses exceeding the permissible limit of 1600 mg/m^2^ would be unacceptable for clothing application due to the concerns about the adverse health effects.

The WHO recommended dosage of permethrin for clothing treatment is 1250 mg/m^2^ [24]. The German military uniforms are treated at the rate of 1300 ± 300 mg/m^2^ and are required to have a minimum concentration of 200 mg/m^2^ to remain effective [17]. The quantification of permethrin from insecticidal fabrics is necessary to ensure that the permethrin dosage is within the accepted range. The treatment method employed should ensure adequate bioavailability of permethrin on the textile surface without causing adverse health effects to the user. Permethrin from the treated fabric is extracted by an organic solvent and chromatographic methods are used to quantify the permethrin content. In the present study, permethrin was extracted in acetonitrile in an ultrasonic bath at 30 °C for 45 min and the quantification was done using HPLC. We have previously reported a HPLC method in which the fabric samples were extracted in acetonitrile. A C-18 analytical column and acetonitrile-water mobile phase were used for permethrin quantification [25]. Another study described HPLC quantification of permethrin wherein the extraction was carried out with acetone in an ultrasonic bath [26]. The HPLC method for permethrin quantification from insecticidal fabrics was reported in two recent studies wherein a C-18 column and water-acetonitrile mobile phase were used [22, 23]. Extraction with toluene in an ultrasonic bath followed by GC estimation was used for permethrin quantification from military uniforms [16, 17, 20]. In another study, high performance thin layer chromatography (HPTLC) was employed to quantify permethrin from military uniforms in which the samples were extracted with acetone and spotted on a silica gel [27].

The fabric samples A and B in the present study contained more than the recommended maximum dose of 1600 mg/m^2^ permethrin, whereas the samples C, D, E and F contained permethrin within the recommended range. In an earlier study, chromatographic estimation showed that the military uniforms polymer coated with permethrin contained 280 mg/m^2^ permethrin even after 100 washings, which was sufficient to provide CKDT of 38.3 min for *Ae. aegypti* [20]. In another study, two permethrin treated civilian fabrics retained only 20 and 40 mg/m^2^ permethrin after 100 washings and the corresponding 99% knockdown times were > 360 and 168 min, respectively, against *Ae. aegypti*. This showed that the fabrics containing permethrin residues lower than the recommended minimum of 200 mg/m^2^ would provide knockdown times exceeding 71.5 min [17]. However, in the present study, continuous exposure to the fabric samples G and H containing 170 and 60 mg/m^2^, respectively, resulted in a CKDT much below 71.5 min. Similarly, another study reported the testing of dipped and factory treated clothes having initial permethrin concentrations of 1250 and 1950 mg/m^2^. After the first washing, the dipped cloth provided 100% knockdown of *Ae. aegypti* on three minutes exposure, whereas the factory treated cloth provided less than 50% knockdown [15]. These studies clearly showed that the bioefficacy of the treated clothes depends on the bioavailability of the insecticide on the fabric surface and hence may not correspond to the permethrin content estimated through chromatographic methods. In the present study, the loss of permethrin from the permethrin dipped fabric after 10 washings was 63.8%. However, factory treatment ensures higher retention of permethrin as revealed by an earlier study wherein the factory treated blankets with an initial permethrin content of 2154 mg/m^2^ lost only 73.8% of permethrin, even after 20 washings [12].

In the continuous exposure method, the sample with the highest permethrin dose of 3000 mg/m^2^ provided CKDT of 10.5 min for *Ae. aegypti* and 14.5 min for *An. stephensi*. The sample fulfilled the bioefficacy criteria as per the improved test method since the CKDT was not more than 71.5 minutes. However, in the three minutes exposure method, the same fabric sample provided KD of only 86% and 76% for *Ae. aegypti* and *An. stephensi*, respectively. The sample would be considered not effective since the minimum KD required is 95%. Similarly, the MR for *Ae. aegypti* and *An. stephensi* (72% and 48%, respectively) were also below the minimum required MR of 80%. Thus, the same fabric sample showed high bioefficacy in the continuous exposure method and no bioefficacy in the three minutes exposure method. Even sample A with the highest permethrin dose (3000 mg/m^2^) failed to qualify the bioefficacy criteria of the three minutes exposure method. Hence, it is clear that insecticidal fabrics with the recommended maximum dose of 1600 mg/m^2^ would fail in the bioefficacy testing using this method. On the other hand, even sample H with the lowest permethrin dose (60 mg/m^2^) passed the bioefficacy criteria as per the continuous exposure method. These results clearly indicated that the outcomes of the continuous exposure method represent the permethrin dosage on the treated fabrics in a better manner. However, further studies are needed to establish the lowest dose of permethrin sufficient enough to achieve a CKDT of ≤ 71.5 min.

The improved test method is derived from the TL 8305-0331 method of the German Armed Forces [16, 17]. The CKDT used in the improved method could be easily determined unlike the 99% KD time used in TL 8305-0331, which requires a series of observations on the KD time and probit analysis [19]. In the improved method, the test outcomes are obtained mostly within 80 min since the KD time of only the fifth mosquito (in a batch of five) is recorded. However, in the three minutes exposure method, a waiting period of 24 h is needed to obtain the percent mortality data. Hence, the new test method is easier to perform and more practically useful for the testing and the quality control of insecticidal fabrics [19].

In a previous study using the three minutes exposure method, we recorded 99% KD and 100% MR of *Ae. albopictus* mosquitoes against permethrin dipped clothes. However, the KD and the MR dropped to 22.9% and 70.5%, respectively, after five washings [25]. The bioefficacy of the unwashed clothes reported here was higher than those observed in the present study for *Ae. aegypti* and *An. stephensi*. This might be attributed to the higher susceptibility of *Ae. albopictus* mosquitoes to insecticidal fabrics in comparison to *Ae. aegypti* and *An. stephensi*. Fabrics with 50% cotton:50% polyester were used in the previous study, whereas 80% cotton:20% polyester were used in the present study. This difference in the fabric composition might be another factor, which affected the bioavailability of the insecticide on the fabric surface, thereby affecting the bioefficacy against mosquitoes. Another study using permethrin dipped cloth (65% cotton:35% polyester) reported 98.3% KD of *Ae. aegypti* mosquitoes [28]. In a study conducted in Thailand, the permethrin treated school uniforms subjected to the three minutes bioassay gave around 100% KD and MR of *Ae. aegypti* mosquitoes initially. The bioefficacy declined rapidly after four washings and was below 20% after 20 washings [3]. The quantification of the permethrin residues was not reported in this study. The recommended minimum dose of permethrin as per TL 8305 0331 is 200 mg/m^2^ [16, 17]. However, the theoretical dosage in the unwashed Thai school uniforms was only 0.054 mg/m^2^. It is unlikely that such low dosage of permethrin would provide almost 100% KD and MR in the three minutes exposure. Hence, a much higher dose might have been actually present on the fabric and bioavailable on the fabric surface. The bioefficacy of insecticidal fabrics depends on the amount of permethrin bioavailable on the fabric surface rather than the total permethrin content present on the fabric [17].

*Aedes aegypti* and *An. stephensi* differ in their levels of susceptibility against insecticidal fabrics as revealed in the present study. In general, *Ae. aegypti* was more susceptible in the continuous and the three minutes exposure bioassays. The CKDT of *Ae. aegypti* was significantly lower than that of *An. stephensi* across a range of permethrin doses indicating higher susceptibility of the former against treated fabrics. Similar results were obtained earlier wherein the factory treated military uniforms washed 100 times provided knockdown times of 38.3 min for *Ae. aegypti*, 44 min for *An. stephensi* and 98 min for *Cx. pipiens* indicating higher bioefficacy against *Ae. aegypti* [17]. This clearly showed that *Ae. aegypti* is the most sensitive and hence the most suitable test mosquito for the evaluation of permethrin treated clothing using continuous exposure methods. Repeated washing and field use might reduce the permethrin content in the treated fabrics to very low levels. Due to their high sensitivity and susceptibility, *Aedes* mosquitoes are ideal test insects for detecting the knockdown and/or mortality effects of low residual amounts of permethrin on a textile surface [17].

Only sample C was used for studying the effects of washing on the bioefficacy, since its permethrin dose (1590 mg/m^2^) was closest to the recommended maximum of 1600 mg/m^2^. The selection of the sample with the highest permissible dose helped us to study the reduction in bioefficacy over 10 successive washings while keeping the permethrin content within the recommended range of (200–1600 mg/m^2^). Continuous exposure of sample C resulted in CKDT of 10.5 min for *Ae. aegypti*. Washing led to a significant decrease in bioefficacy as indicated by the increase in the CKDT up to 48.3 min after 10 washings. The extent of the permethrin loss and the reduction in the bioefficacy depend on the washing procedure followed. In the absence of an internationally accepted standard procedure for the washing of insecticidal fabrics, different methods have been used for the studies on wash resistance. This makes the comparison of the reductions in the permethrin content and bioefficacy recorded in different studies irrelevant [19]. In the present study, the fabrics were washed using the WHO standard washing procedure [21]. In the washing method developed by the Collaborative International Pesticides Analytical Council (CIPAC), the samples are introduced into a glass bottle to which a washing agent is added. The washing and rinsing are carried out by keeping the bottle in a water bath at 30 °C for 10 min each [21]. The testing of permethrin-treated Thai military uniforms was done employing a hand-washing method in which 4 g/l of a household detergent was used. Although around 60% of the initial permethrin content was remaining after three washings, there was no bioactivity observed against *An. dirus* mosquitoes [29]. The residual permethrin from polymer coated insecticidal fabrics dropped from 1300 to 280 mg/m^2^ after 100 cycles of machine washing using a commercially available detergent. The time required to achieve 100% KD of *Ae. aegypti* in continuous exposure tube bioassay was 38.3 min after 100 washings [20]. Another study compared the wash number corresponding to 50% reduction in the mortality (LW_50_) for insecticidal fabrics subjected to WHO standard washing and machine washing. The LW_50_ was 14.4 and 25.6, respectively, with the two washing methods indicated that the WHO method was more rigorous compared to the machine washing [22]. However, the difference in the wash resistance observed in this study could be due to the use of different washing agents. The permethrin treated Thai school uniforms were tested for bioefficacy after hand washing and shade drying. In contrast to the manufacturer’s claim of 70 washings, the bioefficacy rapidly declined after four washings and the KD and the MR were much less than 20% after 20 washings. This showed that the rigorous washing and drying methods adopted in the tropical regions might lead to a higher loss of permethrin than that reported in standard laboratory conditions [3].

The wash resistance of machine washed BDU and civilian fabrics were compared in which the percent retention of permethrin after 100 machine washings was 21.37 in BDU, whereas the percent retention in the civilian fabrics ranged from 1.54 to 41.86 [17]. However, in the present study, the permethrin dipped fabric could retain only 36.2% permethrin after 10 washings and the bioefficacy is unlikely to last up to 100 washings. Apart from the washing method, the method of fabric treatment and the fabric composition might affect the percent retention of permethrin. Polymer coating of permethrin onto fabrics imparts higher wash resistance compared to the dipping method. The polymer recipe and the treatment process need to be optimised in such a way as to provide acceptable levels of wash resistance while ensuring adequate bioavailability of permethrin on the fabric surface after each washing.

## Conclusions

The results of the present study clearly indicated that the bioefficacy testing of the same fabric sample using the continuous exposure and the three minutes exposure methods provided different test outcomes. Consequently, the inferences on the bioefficacy of the test sample based on these two test methods were contradictory to each other. All fabric samples, including those containing doses higher than the recommended maximum, failed to fulfil the bioefficacy criteria in three minutes exposure bioassay, whereas all these samples were effective as per the improved test method. Such a comparative analysis of the two test methods on the same fabric sample is reported for the first time. The mosquito knockdown times recorded in the improved test method correlated better with the permethrin residues in the insecticidal fabrics. Hence, the study indicated that continuous exposure of the test mosquitoes and the estimation of the mean CKDT would be a more appropriate method for the testing of insecticidal fabrics. A bioefficacy cut off value of CKDT ≤ 71.5 min against *Ae. aegypti* mosquitoes and permethrin concentrations of 1300 ± 300 mg/m^2^ initially and ≥ 200 mg/m^2^ after 100 WHO standard washings might be set as the efficacy criteria for the testing of long-lasting insecticidal fabrics. The permethrin extraction and quantification methods employed in the present study did not affect the structural stability of permethrin and these could be recommended for the testing of insecticidal fabrics. The bioefficacy testing and active ingredient quantification of insecticidal fabrics assumes great significance in the context of emergence of diseases such as dengue, chikungunya and Zika spread by mosquitoes. Further studies using different test arthropods are needed to standardise the bioefficacy testing and permethrin quantification of insecticidal fabrics so as to formulate internationally accepted test guidelines.

## Data Availability

All data generated or analysed during this study are included in this published article.

## Abbreviations

LLIN: long-lasting insecticidal nets
WHO: World Health Organization
NMR: nuclear magnetic resonance
HPLC: high performance liquid chromatography
CKDT: complete knockdown time
KD: percent knockdown
MR: percent mortality
ANOVA: one-way analysis of variance
KD_99_: 99 percent knockdown time
HPTLC: high performance thin layer chromatography
BDU: battledress uniform
CIPAC: Collaborative International Pesticides Analytical Council
